# Special Issue—Diabetes Mellitus: Current Research and Future Perspectives

**DOI:** 10.3390/jpm14030308

**Published:** 2024-03-15

**Authors:** Roberto Franceschi

**Affiliations:** Pediatric Diabetology Unit, S. Chiara Hospital of Trento, Azienda Provinciale per i Servizi Sanitari del Trentino (APSS), Largo Medaglie d’Oro, 9, 38122 Trento, Italy; roberto.franceschi@apss.tn.it; Tel.: +39-0461-903538

The Special Issue “Diabetes Mellitus: Current Research and Future Perspectives” is focused on the importance of customized medicine in monogenic diabetes of the young (MODY) and type 2 diabetes (T2D). This collection included five articles that investigated cutting-edge subjects linked to this topic.

Regarding the recent progress in MODY, in this Special Issue, the prevalence of mutations of the gene encoding Hepatocyte Nuclear transcription Factor-1 Beta (HNF1B) was identified in 5.9% of the patients screened, based on the clinical suspicion of MODY. Testing this gene allowed the authors to identify two previously unknown mutations and diverse clinical presentations of HNF1B-related MODY. Multiple extra-pancreatic organ systems could be involved in some forms of MODY, and in HNF1B-related forms kidney involvement was the most frequent extra-pancreatic manifestation [1]. In previous studies, HNF1B mutations in people screened for diabetes and defects of the urogenital tract affected 3 subjects among 210 with MODY [2]. In another study included in this Special Issue, liver lesions, neuropsychiatric symptoms, hyperlipidemia, hyperuricemia, and hypomagnesemia were reported as more prevalent manifestations than renal ones, expanding the understanding of HNF1B-related MODY. It is known that genetic testing plays a crucial role in the implementation of personalized medicine for MODY, as it is essential not only for diabetes treatment and genetic counseling (autosomal dominant inheritance) but also for follow-up timing and modality, as different organ screening must be implemented [3]. However, the classic clinical criteria for MODY diagnosis are often unable to identify all subjects, and using a candidate gene approach leads to missing genetic diagnosis. Therefore, this study sustains the importance of using next-generation sequencing (NGS) panels as a highly sensitive method even for rare forms of monogenic diabetes [4].

In recent years, precision medicine in T2D has focused on pharmacogenomics to provide individualized drug therapy based on a patient’s genetic and genomic information, as various classes of oral hypoglycemic agents are available [5,6]. The papers in this Special Issue address the gaps in the personalized management of T2D complications. Subjects with T2D face higher risks for macro- and microvascular complications than their non-diabetic counterparts [7,8], and precision medicine aims to identify patients who can benefit from a specific treatment more than from others, with the focus of reducing the risk of diabetic complications [9]. Prognostic models can estimate an individual’s risk for relevant complications based on individual risk profiles [9], and different applications are reported in this Special Issue. 

The role of dyslipidemia, which is considered a risk factor for cardiovascular events, has been analyzed through big data analysis using the Code Interpreter plugin of ChatGPT for enhanced predictive modeling. Twelve biochemical parameters were considered in subjects with T2D; HDL was found to be inversely correlated with most of the parameters, and the principal predictors of HDLs were triglycerides, LDL cholesterol, and HbA1c levels. These results suggest that the same approach could be used to derive novel management strategies and therapeutic approaches in T2D [10,11], performing complex analyses with minimal computational and software resources using the available measurements on subjects with T2D and healthy controls. 

A complication of T2D that is under-considered is cognitive dysfunction, as the current management strategies for T2D do not primarily target it, even if many studies have illustrated that T2D increases the risk of cognitive impairment due to impaired insulin signaling, increased oxidative stress, and inflammation [12,13]. In this Special Issue, we included studies that examined the impact of diabetes on cognitive impairment. In India, a high prevalence of cognitive impairment, evident from poor performance in almost all cognitive domains assessed, was detected in subjects with T2D compared with healthy ones. A significant influence of age demographics on cognitive impairment was found, and the association between metformin use and the risk of dementia among people with T2D was investigated. Still, the analysis did not reveal a dose–response relationship between metformin use and incident dementia in T2D patients. These studies suggest that screening for cognitive impairment in people with T2D should be incorporated into routine clinical practice and supported with early treatment through lifestyle and pharmacological interventions [14]. 

Considering another frequent diabetic complication, diabetic foot ulcers, we analyzed the role of personalized treatment, as surgical treatment options include debridement and revascularization [15,16]. In contrast, little research has been conducted on ozone therapy [15,16]. This Special Issue includes a review of recent studies that provides information on ozone therapy in the wounds of patients with diabetic foot ulcers. It led to the conclusion that this therapy is effective, safe, and beneficial, with few adverse effects for treating diabetic foot ulcers. Compared with other treatments, its use accelerates wound healing, which leads to a reduction in costs and hospital stays, and it is contraindicated in only a few conditions [17]. 

In conclusion, with the continuous advancement of new therapies, technology, and cost reduction, personalized medicine is expected to become the mainstream model in the field of MODY and T2D in the future, providing patients with better health management and treatment services.
